# Supplementary material for: A Novel Doppler TRPG/AcT Index Improves Echocardiographic Diagnosis of Pulmonary Hypertension after Pulmonary Embolism
Source: J Clin Med. 2022 Feb 18;11(4):1072. doi: 10.3390/jcm11041072 (PMC8879629; doi:10.3390/jcm11041072)
Supplement: Supplementary file 1 [file jcm-11-01072-s001.zip › jcm-1580754-supplementary.pdf]

Supplementary Table S1. Echocardiographic parameters of 506 PE survivors with functional impairment.

	**PE survivors with FI**		**CTEPH**	**CTED**		**CTEPH or CTED**		**Others**
	**Median (Range)**	**valid N**	**Median (Range)**	**Median (Range)**	**CTEPH vs. CTED**	**Median (Range)**	**Others**	**CTEPH or CTED vs. Others**
Age	66 (19-100)	506	68.5 (24-100)	65.5 (23-86)	ns	68 (23-100)	66 (19-90)	ns
Gender (f%)	F 297 (58%)	506	F 17 (49%)	F 13 (59%)	ns	F 30 (53%)	*F 267 (59%)*	ns
RV 4C	35 (18-67)	382	42 (30-65)	37 (28-50)	**0.008**	39 (28-65)	*35 (18-67)*	**< .001**
AcT (ms)	110 (42-220)	506	80 (42-130)	110 (74-220)	**< .001**	90 (42-220)	*110 (60-200)*	**< .001**
TRPG (mmHg)	25 (3-134)	506	59 (18-134)	25 (14-58)	**< .001**	41 (14-134)	*25 (3-85)*	**< .001**
TRPG/AcT	.223 (0.026-2.197)	506	.718 (.200-2.197)	.278(.100-.703)	<**.001**	.412 (.100-2.197)	*.208 (.026-1.149)*	**< .001**

TRPG-tricuspid regurgitation peak gradient, CTEPH-chronic thromboembolic pulmonary hypertension, CTED-Chronic thromboembolic disease, FI-functional impairment, RV-right ventricle, AcT-pulmonary ejection acceleration time, 4C-four chamber view.
